# Improved Parkinsons disease motor score in a single-arm open-label trial of febuxostat and inosine

**DOI:** 10.1097/MD.0000000000021576

**Published:** 2020-08-28

**Authors:** Hirohisa Watanabe, Tatsuya Hattori, Akito Kume, Kenichiro Misu, Takashi Ito, Yu Koike, Todd A. Johnson, Shigeo Kamitsuji, Naoyuki Kamatani, Gen Sobue

**Affiliations:** a Nagoya University Graduate School of Medicine, Brain and Mind Research Center, Nagoya; b Fujita Health University School of Medicine, Department of Neurology, Toyoake; c Honmachi Clinic, Naka-ku; d KUME Clinic, Mizuho-ku, Nagoya; e MISU Clinic, Miyoshi; f Saishukan Hospital, Kitanagoya, Aichi; g OASIS Clinic, Ushiku, Ibaraki; h StaGen Co., Ltd., Taito-ku, Tokyo, Japan.

**Keywords:** ATP-adenosine triphosphate, clinical trial, hypoxanthine, mitochondria, Parkinson's disease, treatment, xanthine oxidoreductase inhibitors

## Abstract

**Background:**

Cellular energetics play an important role in Parkinsons disease etiology, but no treatments directly address this deficiency. Our past research showed that treatment with febuxostat and inosine increased blood hypoxanthine and ATP in healthy adults, and a preliminary trial in 3 Parkinson's disease patients suggested some symptomatic improvements with no adverse effects.

**Methods:**

To examine the efficacy on symptoms and safety in a larger group of Parkinsons disease patients, we conducted a single-arm, open-label trial at 5 Japanese neurology clinics and enrolled thirty patients (*n*_*males*_ = 11; *n*_*females*_ = 19); 26 patients completed the study (*n*_*males*_ = 10; *n*_*females*_ = 16). Each patient was administered febuxostat 20 mg and inosine 500 mg twice-per-day (after breakfast and dinner) for 8 weeks. The primary endpoint was the difference of MDS-UPDRS Part III score immediately before and after 57 days of treatment.

**Results:**

Serum hypoxanthine concentrations were raised significantly after treatment (Pre = 11.4 μM; Post = 38.1 μM; *P* < .0001). MDS-UPDRS Part III score was significantly lower after treatment (Pre = 28.1 ± 9.3; Post = 24.7 ± 10.8; mean ± SD; *P* = .0146). Sixteen adverse events occurred in 13/29 (44.8%) patients, including 1 serious adverse event (fracture of the second lumbar vertebra) that was considered not related to the treatment.

**Conclusions:**

The results of this study suggest that co-administration of febuxostat and inosine is relatively safe and effective for improving symptoms of Parkinsons disease patients. Further controlled trials need to be performed to confirm the symptomatic improvement and to examine the disease-modifying effect in long-term trials.

## Abstract 2

Supplemental Digital Content is available in the text

## 1 Introduction

Parkinsons disease (PD) is a systemic disease that exhibits not only motor symptoms but also various non-motor symptoms during its course.^[1–3]^ Because of the limitations of L-dopa and other drug therapies, other treatment modalities such as device-aided therapy have been attempted,^[4]^ but at least in the case of deep brain stimulation, several side effects such as voice and speech disorders have been identified as problems.^[5]^ Furthermore, none of the available treatments have been able to modify the course of the disease.

Although the precise mechanisms that lead to PD remain unknown, PD is distinguished by a severe loss of pigmented dopaminergic neurons of the substantia nigra, and alpha-synuclein deposition,^[6,7]^ which is a neuropathological hallmark of sporadic PD.^[8]^ Genetic analyses found that at least 23 loci segregated with or were shown to cause Mendelian/monogenic forms of PD or similar diseases (i.e., those with Parkinsonism as a symptom),^[9]^ and many of the implicated genes had roles related to mitochondrial function or energy metabolism such as alpha-synuclein (*SNCA*; *PARK1*), *PRKN* (*PARKIN*, *PARK2*), *PINK1* (*PARK6*), *PARK7* (*DJ1*), *LRRK2* (*PARK8*), *ATP13A2* (*PARK9*), *CHCHD2* (*PARK22*), and *VPS13C* (*PARK23*).^[10–17]^ In addition, PD genome-wide association studies (GWAS)^[18,19]^ found enrichment for genes related to mitochondrial diseases or mitochondrial function as well as expression in brain cell types related to energy metabolism.^[20,21]^

Observations that patients with neurodegenerative diseases including PD tend to have low uric acid (UA) levels in serum, cerebrospinal fluid (CSF), or brain tissue,^[22–24]^ led to the hypothesis that UA acts as a reactive oxygen species (ROS) scavenger and suppresses their adverse effects. However, the largest test of that hypothesis to date, the Phase 3 clinical trial of the Study of URate Elevation in Parkinsons disease (SURE-PD), examined whether inosine (Ino) administered to PD patients could safely increase blood UA and have a beneficial effect on PD progression,^[25]^ but that trial failed to achieve its primary endpoint.^[26]^ Along with that, the existence of individuals homozygous for genetic xanthine oxidoreductase (XOR) deficiency challenges that hypothesis, since such patients have serum UA levels that are inherently zero but display a complete absence of central nervous symptoms. In fact, XOR is absent or only lowly expressed in the brain,^[27–29]^ the blood brain barrier is minimally permeable to UA, and the concentration of UA in CSF is less than 1/10 of that in the plasma.^[30]^ Therefore, it is unlikely that such low concentrations of UA have major effects on the management of ROS in neuronal cells.

Alternatively, there is a strong hypothesis that neuronal ATP deficiency, such as that which accompanies mitochondrial dysfunction, is involved in PD etiology.^[29,31]^ One facet of ATP deficiency is that UA levels are generally lower in individuals in whom ATP consumption is likely to be decreased, such as infirm elderly or those with low physical functioning or nutritional status.^[32,33]^ Thus, hypouricemia observed in neurodegenerative diseases including PD is possibly due to a decrease in ATP use due to ATP deficiency. In support of that, analyses of brain FDG-PET (Fluorodeoxyglucose-Positron Emission Tomography) to model differences between healthy controls and PD patients identified Parkinsons disease-related patterns.^[34]^ Those patterns were defined by hypometabolism (decreased glucose use) across much of the cerebral cortex (including frontal, premotor, parietal, and occipital regions) and hypermetabolism (increased glucose use) in relatively smaller portions of the brain (parts of the cerebellum, pons, thalamus, and basal ganglia).^[35]^ Since brain glucose is mainly used for ATP regeneration, the larger decrease in glucose metabolism observed in PD patients is likely to reflect a decrease in ATP regeneration. In other words, it suggests that both ATP regeneration and ATP use in the brain are decreased in PD. As further support, studies using the cybrid method identified decreased mitochondrial function in platelets from PD patients, and levels of hypoxanthine (Hyp), the degradation product of Ino via purine nucleotide phosphorylase, were reported to be decreased by 42% in unmedicated PD patients compared to controls.^[36]^

Partly based on such observations, we have recently proposed a hypothesis that a drug combining an XOR inhibitor with additional Ino could enhance cellular ATP.^[29]^ While Hyp can be salvaged back to IMP, which can be converted back to AMP and then to ATP, xanthine (Xan) and UA cannot be reutilized. Inhibition of XOR can be achieved through use of an XOR inhibitor like febuxostat, which was developed to treat gout and hyperuricemia.^[37]^ Treatment with febuxostat would then be expected to decrease degradation of Hyp and lead to greater levels of salvageable Hyp in the body as has been shown in mouse studies.^[38]^ However, metabolic differences exist between mice and humans, and a recent clinical study that we performed suggested that decreased degradation of Hyp is not enough to appreciably raise its level in the blood of human subjects, and that provision of additional purines, in the form of Ino, is needed to achieve increased blood Hyp levels capable of enhancing cellular ATP.^[39]^ In that study, we treated 21 healthy male subjects with febuxostat and/or Ino for 14 days and measured purine compounds in blood or urine. As a result, no or only minor changes of Hyp and ATP in the blood were observed when febuxostat or Ino alone was administered, but by simultaneous administration of 20 mg of febuxostat and 500 or 1,000 mg of Ino twice a day, we observed significant increases of both Hyp and ATP. In addition, we confirmed that treatment with the combined drug was relatively safe.

Based on those findings, 3 patients with PD were co-administered 20 mg of febuxostat and 500 mg of Ino twice a day for 14 days.^[40]^ The results suggested that the treatment may be effective, with 2 cases demonstrating improvement in 2 PD symptoms (decreased tremor and improved mobility) and 1 case showing improvement in 1 PD symptom (decreased tremor); adverse events were not observed (Unpublished data).

Proceeding from that preliminary study, we expanded the number of PD patients, and in the current report, we present results from a study of 30 PD patients who were treated with febuxostat 20 mg and Ino 500 mg twice daily (after breakfast and dinner) for 8 weeks to evaluate efficacy and safety.

## 2 Methods

### 2.1 Study design and participants

The study protocol was approved on December 25, 2017 by Review Board of Human Rights and Ethics for Clinical Studies Ethics Review Committee, Tokyo (http://www.hurecs.org/) and performed in accordance with applicable regulations and guidelines. At 5 Japanese neurology clinics, we performed an uncontrolled (open-label, single-group, multi-center) trial to examine the efficacy and safety of co-administration of febuxostat (Feburic; Teijin Ltd., Tokyo, Japan) and Ino (Inosine; Anabol Naturals, Santa Cruz, CA) to PD patients. This was an investigational use of febuxostat in combination with Ino, and the combined drug is not approved for such use and is still under investigation. Subject enrollment started on January 29, 2018 and drug administration was performed between February 2018 and October 2018. Participating trial sites were Honmachi Clinic, KUME Clinic, MISU Clinic, OASIS Clinic, and Saishukan Hospital. A CRO company, IDD Co Ltd. (Okino Bldg 201, 2-14-19 Minami-Asabu, Minato-ku, Tokyo, Japan), collected and managed the data recorded in the clinics medical charts and made a summary report of the study results that was sent to StaGen Co., Ltd. The validity of the data was checked independently by both the CRO and StaGen Co., Ltd.

Eligible study participants were defined as patients with PD who were diagnosed by specialists in neurology and who fulfilled the study inclusion criteria and did not meet any of the exclusion criteria. PD assessment utilized Hoehn-Yahr scale, Movement Disorder Society-sponsored revision of the Unified Parkinsons Disease Rating Scale (MDS-UPDRS), and Mini-Mental State Exam (MMSE). Inclusion criteria were as follows: Japanese patients who voluntarily participated in the study with written agreement, 20 to 80 years old (as of the date of the written agreement to participate in the study), either male or female, and diagnosed with PD by specialists and satisfied all of the following criteria:

1. Hoehn-Yahr scale is stage 1 to stage 3,
2. MDS-UPDRS part III score is 15 or more,
3. MMSE score is 24 or more.

Patients were excluded if they met any of the following criteria:

1. required nearly full assistance in their daily lives or were incapable of walking and standing,
2. taking azathioprine, mercaptopurine hydrate, vidarabine, or didanosine,
3. serum creatinine exceeded 1.5 times the upper limit of the reference value or patients whose AST (GOT) or ALT (GPT) exceeded 2 times the upper limit of the reference value in the test before the registration,
4. underwent surgical treatment for PD,
5. history or present illness of gout, hyperuricemia, or urolithiasis,
6. past or current treatment with febuxostat,
7. history of hypersensitivity/idiosyncrasy (allergy) to any drug or drugs (not limited to the study drugs),
8. used an investigational drug within 30 days of signing the consent form,
9. pregnant, possibility of pregnancy or lactating, or
10. judged inappropriate for other reasons by the investigator or sub-investigator.

Written informed consent was obtained from each study subject in accordance with the Declaration of Helsinki.

For the study intervention, both a febuxostat 20 mg tablet and an Ino 500 mg capsule were administered orally at the same time with water twice a day after breakfast and dinner. Administration started after dinner on the first day of the study, and the last treatment administration was after breakfast on the 57th study day. Subjects were advised that if they forgot to take the medication, that they should not take 2 doses at once, and that they should take the medicine according to the usage and dose from the next scheduled time. Drugs other than the study drugs were permitted, but change of usage and dose was not permitted during the test period.

This trial was registered on January 22, 2018 with the UMIN Clinical Trials Registry (https://www.umin.ac.jp/ctr/) as UMIN000030930.

### 2.2 Study outcomes

MDS-UPDRS was used to score the level of PD symptoms.^[41,42]^ MDS-UPDRS Part III was scored while the subjects were in the “ON” state. All of the investigators were specialized in clinical neurology. Those without the “Certificate Exercise” for MDS-UPDRS training from the Movement Disorder Society (https://www.movementdisorders.org/) underwent the MDS-UPDRS Training Program before the start of this study under the permission of the International Parkinson and Movement Disorder Society.

Additional endpoints came from laboratory measurements performed on whole blood and plasma samples collected on the 1st, 29th, and 57th days of the study. Standard clinical laboratory tests including total protein (TP), total cholesterol (T-Cho), UA, and red blood cell count were performed by commercial clinical laboratory test companies that routinely perform the tests for the clinics. Additionally, measurements of Hyp and Xan in the peripheral blood were made using previously published methods.^[39,43]^ Briefly, we collected peripheral blood into an EDTA-2A blood collection tube and then mixed 500 μl of blood with the same amount of ice cold 8% perchloric acid (PCA). The mixture was then immediately vortexed, after which, it was centrifuged at 12,000× *g* for 5 seconds at 4 C and the supernatant then stored at −80 C. Prior to analysis, we added 40 μl of 2 M K_2_CO_3_ in 6 M KOH to 650 μl of the lysate. That was then centrifuged at 12,000× *g* for 10 minutes at 4 C. Then, 160 μl of mobile phase was added to 40 μl of supernatant and run on a high-performance liquid chromatography (HPLC) instrument. HPLC conditions were as described by Fukuuchi et al.

### 2.3 Primary endpoint

The primary efficacy endpoint of the study was the MDS-UPDRS Part III score change from before the start of treatment to the 57th study day.

### 2.4 Secondary endpoints

1. MDS-UPDRS Part III score change from before the start of the medication to the 29th day of the medication.
2. MDS-UPDRS (Part I, Part II, Part IV) score change from before the start of the medication to the 57th day of the medication.
3. MDS-UPDRS (Part I, Part II, Part IV) score change from before the start of the medication to the 29th day of the medication.
4. Subjective evaluation by the patient (or care giver) of the severity of the original disease before and after administration (57th day of administration of the test drug)
5. Subjective evaluation by the patient (or care giver) of the severity of the original disease before and after treatment (the 29th day of administration of test drug)
6. Examination of the severity of the original disease before and after treatment by the investigator or the sub-investigator doctor (57th day of administration of the test drug)
7. Examination of the severity of the original disease before and after treatment by the investigator or the sub-investigator doctor (29th day of administration of the test drug)
8. Changes in general laboratory test values (TP, T-Cho, red blood cell count, Hyp, Xan, UA) from 1st day (before start of administration) to the 57th day
9. Changes in general laboratory test values (TP, T-Cho, red blood cell count, Hyp, Xan, UA) from 1st day (before start of administration) to 29th day.

### 2.5 Protocol rationale and statistical analysis

The approved maximum dose of febuxostat in Japan is 60 mg once a day. In this study, 20 mg of febuxostat and 500 mg of Ino were administered twice a day after breakfast and dinner. That modification was made based on previous pharmacokinetic/pharmacodynamic data^[44]^ and had the goal of maintaining high blood levels of Hyp and ATP throughout the day. The 8 weeks administration period was deemed a sufficient period to evaluate efficacy and safety in an early-stage clinical trial of PD. It was expected to be sufficient to evaluate the symptomatic improvement of the disease although not sufficient to evaluate the disease-modifying effect.^[45]^

Microsoft Excel 2010 was utilized for data collection, and R version 3.5.1^[46]^ was used for statistical analyses. Study sample-size to achieve 80% power at α = 0.05 could be calculated using standard methods^[47]^ for a two-sided paired *t* test such as is implemented in the pwr.*t*.test function in the R *pwr* package^[48]^ with standard deviation (SD) and target mean approximated from previous PD intervention study results. For example, estimates of the standard deviation (SD) of the change in MDS-UPDRS Part III score after 12 months of treatment was available in the SURE-PD report (SD_Mild_ = 3.42; SD_Moderate_ = 6.12),^[25]^ while a suitable score change mean target was previously described as the MDRS-UPDRS Part III minimal clinically important difference (MCID = −3.25).^[49]^ From those values, study sample-size estimates ranged from 10.8 to 29.8, and target enrollment was set at 30 cases as the number of cases that even with some amount of study subject dropout would likely be able to verify a change of the primary efficacy endpoint (MDS-UPDRS Part III) between the trial start and end dates.

In the text, figures, and tables, primary and secondary endpoint data are summarized as mean ± SD unless otherwise noted. Changes in primary and secondary endpoints mean values were analyzed using two-tailed paired *t* tests. Use of that test was stipulated in the study protocol, but to confirm the result using a non-parametric analysis, we also performed a Wilcoxon signed-rank sum test. Subjective evaluation of post-treatment disease severity by patients or physicians was analyzed using a two-tailed t test with the null hypothesis that the mean value was zero.

## 3 Results

**Figure 1:**
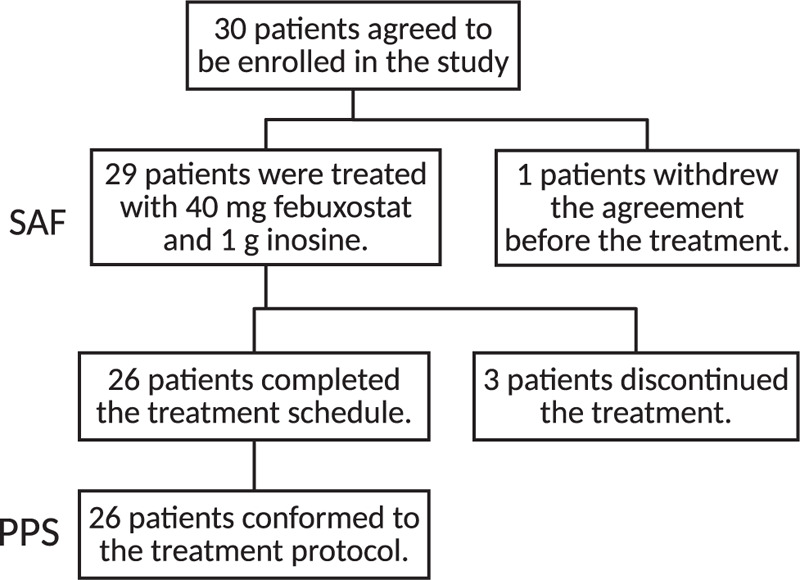
Trial participant flow diagram.

Thirty patients were enrolled in the study, but 1 patient withdrew consent before the start of the treatment. The 29 patients who started treatment ranged in age from 50 to 79 years old (Subject counts by age range: 50–59 y.o. = 6, 60–69 y.o. = 8, 70–79 y.o. = 15), and the treatment was discontinued in 3 patients and it was completed in the remaining 26 patients. Safety was assessed in the Safety Analysis Set (SAF) of 29 patients, and the efficacy was assessed in the Per Protocol Set (PPS) of 26 patients (Fig. 1). Patient demographics are shown in Table 1.

**Table 1:**
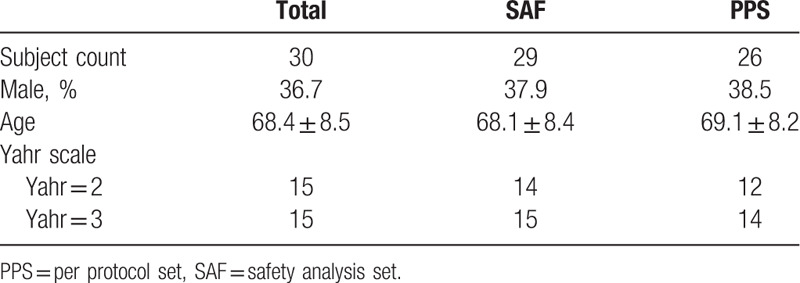
Subject demographics.

**Figure 2:**
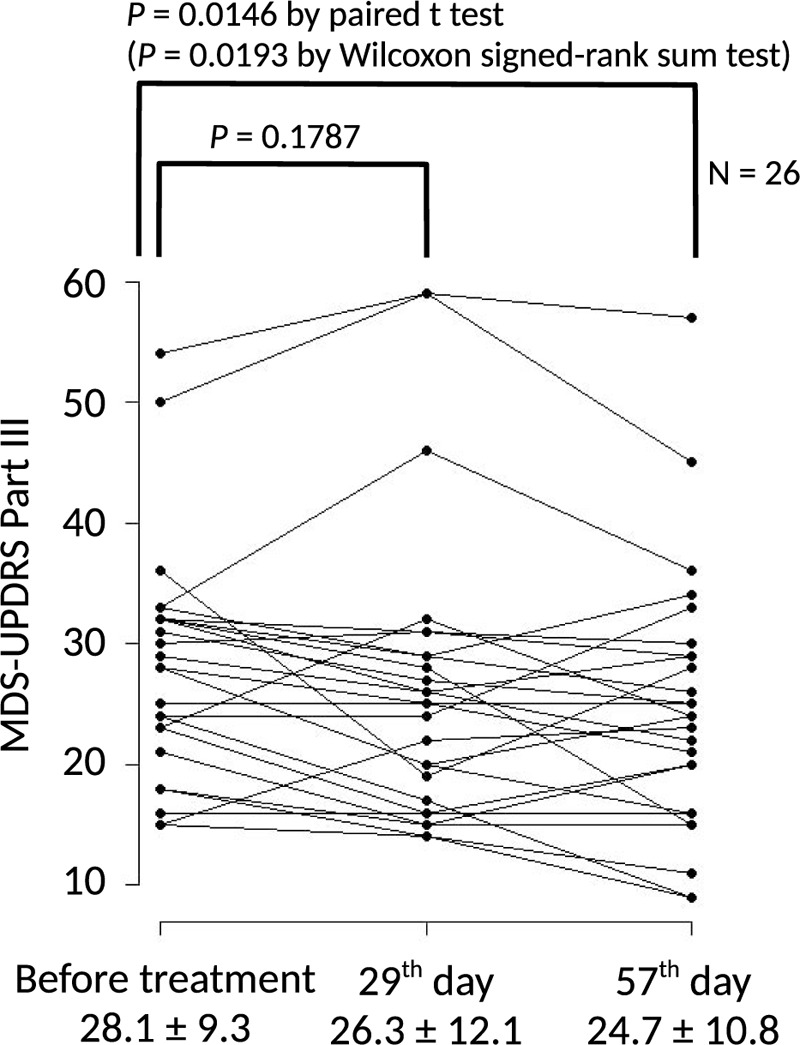
Pre- and post- treatment MDS-UPDRS Part III scores. Graph showing the change of MDS-UPDRS Part III score in each of the 26 patients. Primary endpoint was the change of MDS-UPDRS Part III score from before beginning treatment to 57th day; significance was calculated using a paired t-test with 25 degrees of freedom. Results of the Wilcoxon signed-rank sum test are presented as an alternative non-parametric method. Values below the plot represent mean ± SD of the MDS-UPDRS Part III scores.

The primary endpoint, MDS-UPDRS Part III score, decreased significantly from before the treatment to the 57th day of the medication (Pre = 28.1 ± 9.3; Post = 24.7 ± 10.8; mean ± SD; *P* = .0146 by paired t test) (Fig. 2; Table 2). The change of the mean value was −3.4, which fulfilled a previously established criteria for the minimal clinically important difference for MDRS-UPDRS Part III score of −3.25.^[49]^ Analysis using the Wilcoxon signed-rank sum test also showed a significant difference of *P* = .0193.

**Table 2:**
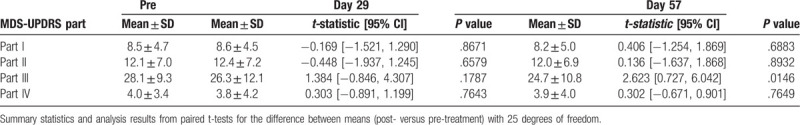
MDS-UPDRS results.

In the secondary endpoint analysis, although Part III scores decreased between the start of medication and the 29^th^ day of treatment, the difference was not significant (Fig. 2; Table 2), and scores for MDS-UPDRS Parts I, II, and IV were not significantly different between the 29th or 57th day and the pre-treatment values (Table 2). Subjective evaluation of change in disease severity with treatment was performed by patients (or care-givers) as well as the study's physician investigators (or sub-investigators) and the analysis shown in Table 3. Patients evaluated that disease severity had significantly improved by the 57th day (*P* = .0056) but not at the 29th day (*P* = .2649). In contrast, the physicians had evaluated that disease severity had improved by both the 29th (*P* < .0001) and 57th days (*P* = .0012).

**Table 3:**
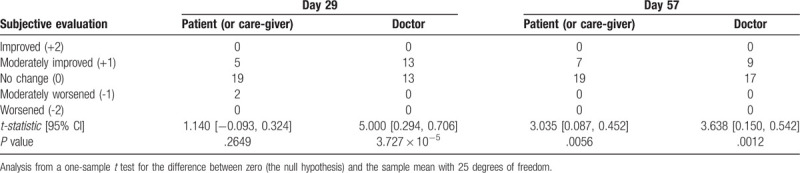
Subjective evaluations by patients (or care-givers) and doctors.

There was no significant difference in general laboratory values (TP, T-Cho and red blood cell count) between day 1 and either the 29th or 57th day, but at both timepoints, Hyp and X were significantly increased, while UA was significantly decreased (*P* < .0001 for each comparison) (Table 4). Systolic blood pressure, diastolic blood pressure, pulse rate, and body temperature did not change between before the treatment and either the 29th or 57th day of the treatment period (data not shown).

**Table 4:**
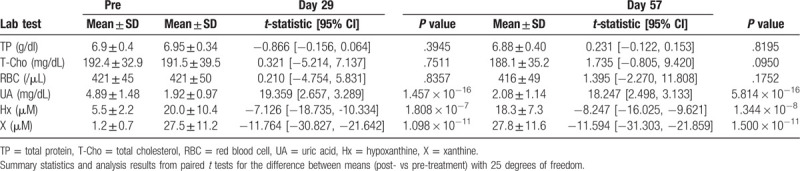
Blood laboratory results.

A total of 16 adverse events occurred in 13 (44.8%) patients including 1 serious adverse event (fracture of the second lumbar vertebra) that was considered not related to the treatment (Table 5). In addition to the fracture of the second lumber vertebra stated above, adverse events included anxiety, hypotension, nail Trichophyton infection, constipation, feeling of swelling in the lower limbs, worsening of chronic gastritis, hallucination, floating dizziness, atheroma of the buttock, and intestinal disorder. In 3 patients, the treatment was discontinued, with discontinuation due to adverse events in 2 patient's and due to the patient's personal reasons in the other.

**Table 5:**
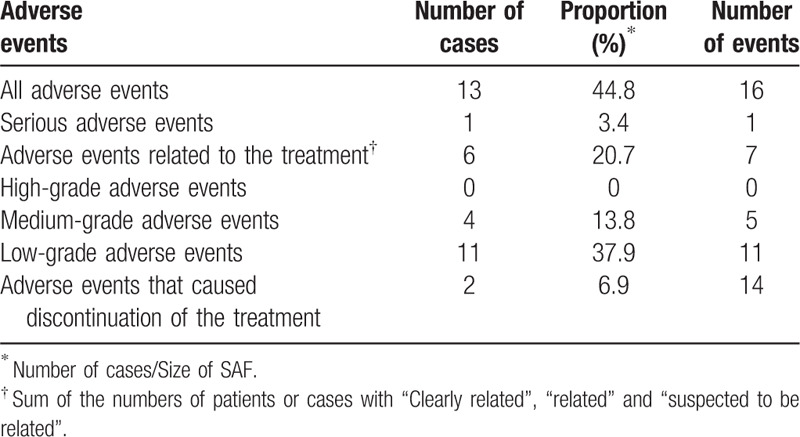
Adverse events summary.

To further examine the potential impact of adverse events on subjective evaluations, we tabulated adverse event status versus patient and doctor assessments on Day 29 and Day 57 (Table S1 Supplemental Digital Content, which tabulates subjective evaluations by patients and doctors by the most severe adverse event experienced by a patient) and performed a statistical analysis of the between group differences. While both patient and doctor evaluations tended to be higher (showed greater improvement) for patients who had not experienced an adverse event compared to those who had, we found no significant differences between those groups for any of the subjective evaluation results (Table S2 Supplemental Digital Content, which analyzes whether subjective evaluations by patients or doctors differed if a patient had had an adverse event).

## 4 Discussion

Although alpha-synuclein deposition has become a recent target of PD drug development,^[50]^ PD patients with the most common inherited forms, those with mutations in Parkin (*PRKN*) or leucine rich-repeat kinase 2 (*LRRK2*), sometimes lack such alpha-synuclein aggregates.^[51,52]^ In addition, a large proportion of individuals possessing alpha-synuclein deposits do not exhibit clinical symptoms of PD or other dementia related disorders.^[53]^ In light of the strong overlap of PD-related genes with mitochondrial function, including alpha-synuclein itself,^[17]^ such discrepancies have led to a hypothesis that mitochondrial dysfunction plays a role in the disease's etiology.^[8]^

The hypothesis that mitochondrial dysfunction causes cell death in neurodegenerative diseases has been proposed previously,^[54,55]^ and it is well known that in physiological conditions, the brain consumes large amounts of energy since neurons have to operate Na/K-ATPases to maintain the sodium and potassium gradients across cell membranes, incorporate neurotransmitters to synaptic vesicles, move synaptic vesicles to the synaptic terminal, and recover neurotransmitters back from the synaptic space. Therefore, it is likely that energy shortage causes the dysfunction of the cells before cell death occurs.

To ameliorate the effects of such mitochondrial dysfunction and enhance cellular ATP levels, we recently proposed the use of an XOR inhibitor and Ino as a combination drug,^[29]^ and in our recent clinical study, we showed that both blood Hyp and ATP increased in healthy volunteers after 2 weeks of such treatment.^[39]^ One possible question concerns whether increasing Hyp levels in the blood in such a manner can lead to increased purine salvage in the brain. Evidence for that can be found in animal studies that demonstrated the existence of transport mechanisms by which Hyp can cross the blood brain barrier and the choroid plexus,^[56–58]^ and in a human study of patients with hypoxanthine-guanine phosphoribosyltransferase (*HPRT1*) deficiency, in which allopurinol treatment led to 5X and 2X increases of Hyp levels in the blood and cerebral spinal fluid, respectively.^[59]^ A more recent report identified SLC43A3 as an equilibrative nucleobase transporter and showed that it acts in concert with HPRT1 to convert Hyp to IMP upon transport across the cell membrane.^[60]^ Importantly, its expression was found to localize to microvasculature endothelium such as that present in brain microvessels.^[61,62]^ Therefore, SLC43A3 may be responsible for the down-gradient transport of Hyp from blood to CSF that was observed in Jimenez et al.

Our present uncontrolled study showed that treatment of PD patients with the XOR inhibitor febuxostat and Ino for 2 months improved MDS-UPDRS Part III scores significantly (*P* = .0146). Furthermore, the change of the mean value of −3.4 fulfilled a threshold of −3.25 that was previously identified for detecting minimal clinically relevant improvement from MDRS-UPDRS Part III scores.^[49]^ Subjective evaluations by both patients and doctors found that disease severity was significantly improved by the 2-months treatment (*P* = .0056 and *P* = .0012, respectively), with the doctors’ assessments also noting significant improvement by 1-month of treatment (*P* = 3.7 × 10^–5^). Serum Hyp was markedly elevated after both 1 month and 2 months of treatment (*P* < .0001). That Hyp was elevated at both timepoints but that heterogeneity was observed in the improvement of efficacy measures at 1 month versus 2 months of treatment suggests that the treatment effect may be related to the duration of time over which increased Hyp levels occur. In addition, the analysis showed that the treatment was relatively safe. These data suggest that the treatment with simultaneous administration of febuxostat and Ino or the mixed drug containing the 2 compounds may be useful for PD.

In addition, we also recently showed that the same treatment improved the laboratory data of 2 mitochondrial disease patients, one with mitochondrial cardiomyopathy and the other with mitochondrial diabetes, and brain natriuretic peptide (BNP) decreased by 31% in the former patient, and in the latter, insulinogenic index increased by 3.1 fold.^[63]^ Taken together, these data suggest that our treatment may improve disorders induced by cellular ATP shortage, which can be considered a reason for the abnormalities found in mitochondrial disease patients.^[64]^

The results of this study suggest that co-administration of febuxostat and Ino may be safe and effective for improving symptoms of PD patients. Further controlled trials need to be performed to confirm the symptomatic improvement and to examine the disease-modifying effect in long-term trials.

## Author contributions

**Conceptualization:** Naoyuki Kamatani.

**Data curation:** Shigeo Kamitsuji.

**Formal analysis:** Shigeo Kamitsuji.

**Investigation:** Hirohisa Watanabe, Tatsuya Hattori, Akito Kume, Kenichiro Misu, Takashi Ito, Yu Koike, Gen Sobue.

**Project administration:** Hirohisa Watanabe, Gen Sobue.

**Resources:** Hirohisa Watanabe, Akito Kume, Kenichiro Misu, Takashi Ito, Yu Koike, Gen Sobue.

**Supervision:** Hirohisa Watanabe, Gen Sobue.

**Visualization:** Todd Andrew Johnson, Naoyuki Kamatani.

**Writing – original draft:** Todd Andrew Johnson, Naoyuki Kamatani.

**Writing – review & editing:** Hirohisa Watanabe, Tatsuya Hattori, Akito Kume, Kenichiro Misu, Takashi Ito, Yu Koike, Todd Andrew Johnson, Shigeo Kamitsuji, Naoyuki Kamatani, Gen Sobue.

## Supplementary Material

Supplemental Digital Content

## Supplementary Material

Supplemental Digital Content
